# Involvement of the Extrageniculate System in the Perception of Optical Illusions: A Functional Magnetic Resonance Imaging Study

**DOI:** 10.1371/journal.pone.0128750

**Published:** 2015-06-17

**Authors:** Ken-ichi Tabei, Masayuki Satoh, Hirotaka Kida, Moeni Kizaki, Haruno Sakuma, Hajime Sakuma, Hidekazu Tomimoto

**Affiliations:** 1 Department of Dementia Prevention and Therapeutics, Graduate School of Medicine, Mie University, Mie, Japan; 2 Department of Neurology, Graduate School of Medicine, Mie University, Mie, Japan; 3 Faculty of Medicine, Mie University, Mie, Japan; 4 Department of Radiology, Graduate School of Medicine, Mie University, Mie, Japan; University of Toyama, JAPAN

## Abstract

Research on the neural processing of optical illusions can provide clues for understanding the neural mechanisms underlying visual perception. Previous studies have shown that some visual areas contribute to the perception of optical illusions such as the Kanizsa triangle and Müller-Lyer figure; however, the neural mechanisms underlying the processing of these and other optical illusions have not been clearly identified. Using functional magnetic resonance imaging (fMRI), we determined which brain regions are active during the perception of optical illusions. For our study, we enrolled 18 participants. The illusory optical stimuli consisted of many kana letters, which are Japanese phonograms. During the shape task, participants stated aloud whether they perceived the shapes of two optical illusions as being the same or not. During the word task, participants read aloud the kana letters in the stimuli. A direct comparison between the shape and word tasks showed activation of the right inferior frontal gyrus, left medial frontal gyrus, and right pulvinar. It is well known that there are two visual pathways, the geniculate and extrageniculate systems, which belong to the higher-level and primary visual systems, respectively. The pulvinar belongs to the latter system, and the findings of the present study suggest that the extrageniculate system is involved in the cognitive processing of optical illusions.

## Introduction

The neural processing of an optical illusion can provide clues for understanding the neural mechanisms underlying visual perception. To study form perception and object recognition, optical illusions such as illusory contours or geometric illusions are often used [1–5].

There are two types of optic pathways: the geniculate and extrageniculate pathways [6–8]. The geniculate visual pathway involves transmission from the retina to the lateral geniculate nucleus (LGN) and then to the visual cortex. In contrast, the extrageniculate visual pathway runs from the retina to the superior colliculus, the pulvinar, and finally the visual cortex.

Some studies have reported that the neural mechanisms underlying processing of optical illusions occur in the early stage of visual processing, especially because neural activity related to this processing has been found in V1/V2 [9–14]. For instance, Murray et al. [12] demonstrated that the retinal size of an object and the depth information in a scene are combined in V1 and size illusions are reflected in the spatial pattern of activity in V1. Regarding the role of V2, Ramsden et al. [10] reported that the abutting line grating illusory contour activates orientation domains in V2 that overlap with those activated by luminance gratings.

However, other studies have demonstrated that the processing of optical illusions also depends on higher stages of visual processing. The lateral occipital cortex (LOC), which is highly involved in object recognition, has been shown to contribute to the processing of optical illusions [2, 3, 15–18]. Studies on patients with brain damage support this finding [1, 19]. Although these were not studies of individuals with brain damage, Daini et al. [19] revealed an association between damage to the occipital regions and the inability to perceive illusory effects. Patients with neglect and left hemianopia showed a lack of perception of illusory effects and had large bisection errors. These findings suggest that optical illusion processing likely occurs in the occipital cortex, at a retinotopic level of representation. From these previous studies, it can be concluded that the neural processes underlying the perception of optical illusions occur in the occipital cortex, specifically in the LOC.

In actuality, both early and higher-level visual stages are necessary for processing an optical illusion; the illusory aspect depends on the interaction between different levels of visual processing [20–22]. Feedback processing has been suggested to occur during optical illusion processing, that is, the processing of an optical illusion may follow an inverse hierarchical path [23–27]. Wokke et al. [28] used transcranial magnetic stimulation to disrupt signaling in the V1/V2 and LOC at different time points while participants performed a discrimination task for an illusory figure. Their results demonstrated that both V1/V2 and the LOC are critically involved in the perception of an optical illusion. These areas seem to be related in an inverse hierarchical fashion such that the critical time window for V1/V2 follows that for LOC. It was therefore suggested that after initial perceptual processing in the early visual cortices, optical illusions are detected by a higher visual area. Then, the information is fed back to the early visual cortices for completion, with strengthening of the figure-ground segregation processes or reception of predictive signals from higher visual areas.

However, these previous reports on the processing of optical illusions have the following limitations: (1) the visual stimuli differed between the main and control tasks, (2) the optical illusion figures were almost always restricted to the Kanizsa type and Müller-Lyer figure, and (3) the neural processing of the control stimuli was not always clarified. Therefore, the major aim of the present study was to examine the neural mechanisms underlying the processing of optical illusions using various illusory figures. We hypothesized that there are common mechanisms that account for line (e.g., Müller-Lyer) and area (e.g., Kanizsa) types of optical illusion. In previous studies, the use of different stimuli for the main and control tasks may have led to differences in the activated brain regions. Therefore, as suggested by previous studies, the question of whether or not the information from higher levels is fed back to early visual cortices remains, and there could be an alternative explanation that suggests involvement of extrageniculate pathways. Therefore, we performed functional magnetic resonance imaging (fMRI) during the presentation of optical illusions using Müller-Lyer, Ponzo, Hefler, Zerbino, Ebbinghaus, Jastrow, and Delboeuf figures. These figures consist of many kana letters, which are Japanese phonograms. We used the same stimuli for both the main task and the control task to detect differences in cognitive processing.

## Materials and Methods

### Participants

Eighteen right-handed volunteers (9 men, 9 women; mean age, 21.9 ± 1.2 years) participated in this study. All participants had normal or corrected-to-normal vision. The participants provided written informed consent before the experiment in accordance with the Declaration of Helsinki. Our study was approved by the ethics committee of Mie University.

### Stimuli

**Fig 1:**
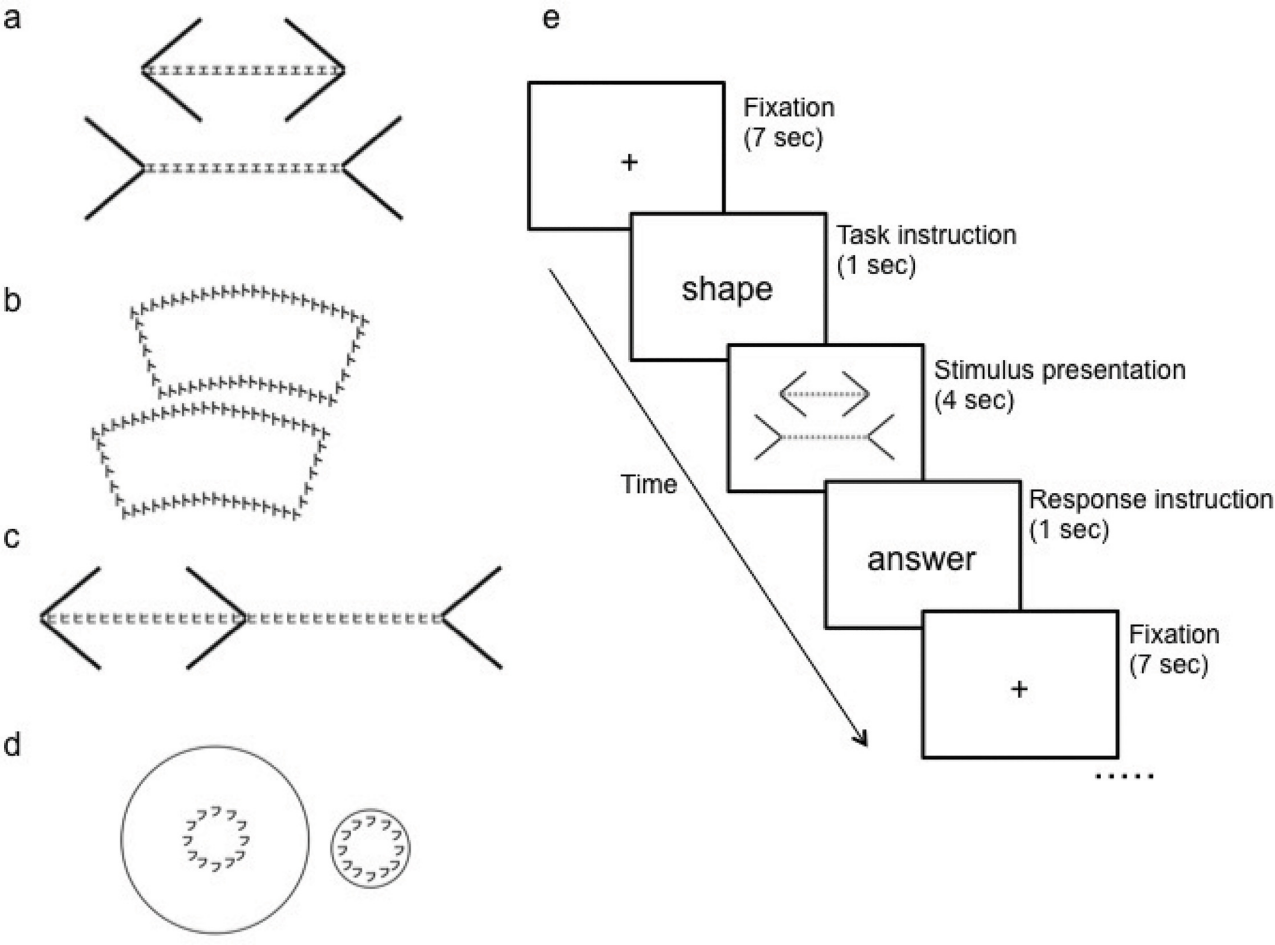
The stimuli in the present experiment were illusory figures generated from many kana letters, which are Japanese phonograms. For example: (a) Müller-Lyer illusion with the Japanese phonogram “e,” (b) Jastrow illusion with the Japanese phonogram “to,” (c) Müller-Lyer illusion with the Japanese phonogram “hi,” and (d) Delboeuf illusion with the Japanese phonogram “hu.” Representation of the examples of a trial time course (e). Each stimulus appeared for 4 s, with an interstimulus interval of 9 s.

The stimuli used in the present experiment were illusory figures consisting of many kana letters, which are Japanese phonograms (e.g., Fig 1A–1D). Each stimulus was adopted from two books of optical illusions [29, 30]. We changed the line of the stimuli to Japanese phonograms. We used the following types of illusions: 2 types of Müller-Lyer illusions with the Japanese phonogram “e” or “hi”; the Ponzo illusion with “na”; 2 types of Hefler illusions with “sa” or “ki”; the Zerbino illusion with “he” as an optical illusion of lineal length; 3 types of Ebbinghaus illusions with “ka,” “mi,” or “su”; 2 types of Jastrow illusions with “to” or “re”; and the Delboeuf illusion with “hu” as an optical illusion for area. Each stimulus was displayed in two different ways by flipping it vertically or horizontally. Thus, we used 12 types of stimuli and there were 24 total stimuli. Stimuli were displayed using magnetic resonance (MR)-compatible goggles (CinemaVision, Resonance Technology Inc., CA) at a total resolution of 240,000 pixels and covered a visual angle of 30° horizontally and 22.5° vertically. The actual size of the stimulus was 630 × 473 pixels. Black stimuli were presented in the center of the screen on a white background. Stimuli were controlled using E-Prime software (Psychology Software Tools, Inc., PA) on a personal computer. There was no modulation of stimulus size across trials.

### Tasks

Our experimental design consisted of two experimental tasks, namely a shape and a word task. During the shape task, a stimulus was presented and participants were instructed to judge whether they perceived the length/area constituted by the Japanese letters of two optical illusions as being the same or not, and to say their answer aloud. During the word task, the participants saw the stimulus and read aloud the kana letters present. Participants were instructed to respond as quickly as possible on all trials at the response instruction. During the baseline periods, a fixation cross was presented. Participants practiced the tasks both inside and outside of the scanner until they could execute them confidently with specific training material before the experiment.

### Session

A mixed design containing features of both blocked and event-related approaches was used. Each condition (word/shape task) lasted for 39 s, including the instruction slides, followed by a baseline period (20 s). When contrasts were calculated against the baseline, the baseline was thus implicitly defined as a rest block of 20 s (mean signal during non-modeled periods). During each condition, 3 trials/stimuli were presented in random order every 13 s; each stimulus appeared for 4 s, with an interstimulus interval of 9 s (Fig 1E). Each stimulus was presented with equal frequency. This experiment was composed of 2 sessions per participant, and each condition was presented 8 times in random order per session.

### fMRI measurements

All images were acquired using a 3.0-Tesla MR scanner (Achieva Quasar dual 3.0-Tesla, Koninklijke Philips Electronics). Functional images were obtained using a T2*-weighted gradient-echo echo planar imaging sequence (repetition time [TR] = 3000 ms, echo time [TE] = 35 ms, flip angle = 90°, slice thickness = 5 mm, gapless, field of view [FOV] = 240 mm, 96 × 96 matrix). The voxel size was 2.5 * 2.5 * 5 mm^3^. In addition, a T1-weighted anatomical image was obtained for each subject (TR = 7.6 ms, TE = 3.6 ms, flip angle = 8°, slice thickness = 0.7 mm, FOV = 250 × 250 mm, in-plane resolution = 1.04 × 1.04 mm).

### fMRI data analysis

Preprocessing and data analysis were performed using SPM8 software (Wellcome Department of Imaging Neuroscience, London, UK). The first five functional images were discarded to account for magnetic saturation. In total, 319 images were acquired per session and per subject. The functional images were temporally corrected for acquisition time differences with regard to the middle slice, realigned to the first image to correct for movement-related effects, coregistered to the anatomical image, normalized to the Montreal Neurological Institute (MNI) brain template, and spatially smoothed with an isotropic Gaussian kernel (full width at half maximum = 8 mm). We conducted voxel-wise statistical analyses based on the general linear model. For the statistical model, an event-related design was modeled using the canonical hemodynamic response function and temporal derivative, and low-frequency drifts were removed using a high-pass filter (128 s). The onsets were defined as the onset time of the stimulus presented. For each participant, we computed contrasts for “shape task > baseline,” “shape task > word task,” “word task > baseline,” and “word task > shape task.” A random-effects model was used for the group analysis. We used two different correction levels: voxel-level correction was employed for baseline contrasts and cluster-level correction for differential contrast. We assessed the statistical significance at a single voxel threshold of *p* < 0.05, family-wise error [FWE]-corrected (voxel-level corrected) or cluster threshold of *p* < 0.05, FWE-corrected with a voxel threshold of *p* < 0.001, uncorrected (cluster-level corrected), and activations that involved a contiguous cluster of at least 10 voxels were reported. MNI coordinates indicating the peak activation were converted to Talairach coordinates [31] using a non-linear transformation of the MNI brain image to the Talairach brain image (http://imaging.mrc-cbu.cam.ac.uk/imaging/MniTalairach). The active cortical areas were found using Talairach Client [32].

### Effective connectivity analysis

In the analysis, we focused on the function of the pulvinar and visual cortex, which are part of the geniculate and extrageniculate optic pathways to investigate whether the extrageniculate system is involved in the cognitive processing of optical illusions. The geniculate visual pathway involves transmission from the retina to the lateral geniculate nucleus (LGN) and from there to the visual cortex. In contrast, the extrageniculate visual pathway starts from the retina and includes the superior colliculus, the pulvinar, and finally the visual cortex. First, volumes of interest (VOIs) were selected, based on activity and anatomical constraints [6–8]. V1 was defined as an area showing greater activation for shape and word tasks compared to the baseline. The pulvinar was defined as an area showing greater activation for the shape task compared to the word task. VOI selection was based on the T-contrasts adjusted with the F-contrasts (*p* < 0.01 uncorrected). VOIs were spherical, with a radius of 8 mm around the peak activation. The variance explained by the first eigenvariate of the blood oxygenation level dependent signals was higher than 69%.

The effective connectivity was tested by DCM-10, implemented in SPM8 toolbox (Wellcome Department of Imaging Neuroscience, London, UK). Models of the DCM were defined with endogenous connections, representing coupling between brain regions (matrix A), modulatory connections (matrix B), and driving input (matrix C). In matrix A, we defined the connections between V1 and the pulvinar. In matrix B, we defined the modulatory connections from V1 to the pulvinar or from the pulvinar to V1. Images of illusory figures consisting of many kana letters, i.e., Japanese phonograms, served as the driving input (matrix C).

**Fig 2:**
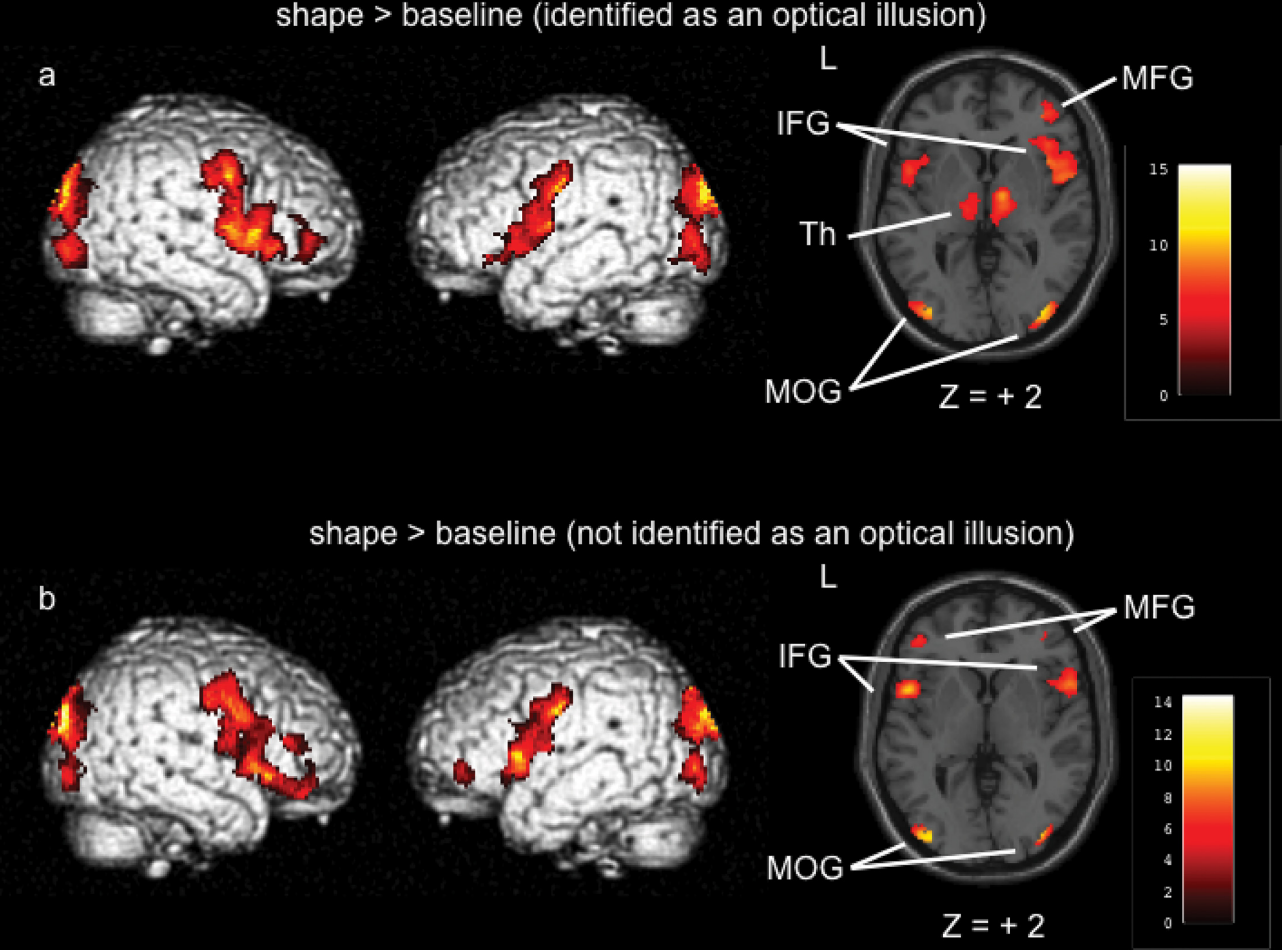
The four analyzed models having different matrix A structures (a). For details, see the “Materials and Methods” section. Results of Bayesian model selection of the random effects design on the model levels. Model expected probability (b) and model exceedance probability (c). p = pulvinar.

Model estimation was conducted to maximize the negative free-energy estimates of the models (F) for a given dataset [33]. Furthermore, Bayesian model selection (BMS) was conducted for random (RFX) effect designs. In Model 1, visual information was input to the pulvinar, and the shape task, as a modulatory input, had an effect on the forward connections from the pulvinar to V1. Model 1 represents blindsight, which is the ability to respond to visual stimuli without consciously seeing them and is exhibited by people with cortical blindness resulting from lesions in the primary visual cortex. In Model 2, visual information was input to V1, and the shape task, as a modulatory input, had an effect on the forward connections from V1 to the pulvinar. In Model 3, visual information was input to V1 and the pulvinar, and the shape task, as a modulatory input, had an effect on the forward connections from the pulvinar to V1. In Model 4, visual information was input to V1 and the pulvinar, and the shape task, as a modulatory input, had an effect on the forward connections from V1 to the pulvinar (Fig 2A). The four models were compared by a random-design BMS to identify the model with the highest exceedance probability.

## Results

### Behavioral data

Fourteen sessions were excluded because the rate at which a stimulus was identified as an optical illusion was below the level attributed to chance (50%: true or false). Thus, we used 22 sessions in the final analysis. The identification rates for the optical illusions were 77% and 73% for the shape task with lines and the shape task with areas, respectively. The rates of identification for word stimuli were 98% and 98% for the word task with lines and the word task with areas, respectively. We performed a task (shape or word) × stimulus type (line or area) mixed-factor analysis of variance (ANOVA) on the stimulus identification rates for optical illusions or words. There was no significant main effect of stimulus type [*F* (1, 21) = 0.851, *p* = 0.37] or interaction between task and stimulus type [*F* (1, 21) = 0.296, *p* = 0.59]. However, there was a significant main effect of task [*F* (1, 21) = 71.05, *p* < 0.001]. Because reading single kana letters is an easy task, the behavioral results indicate that the identification rate for word stimuli was higher than that for optical illusions (Table 1).

**Table 1:** Identification rates for each figure/stimulus (all sessions).

			Identification rates (%)	
illusion	word	type	shape task	word task
Muller-Lyer	エ (e)	line	64	100
Muller-Lyer	エ (e)	line	64	100
Ponzo	ナ (na)	line	77	100
Ponzo	ナ (na)	line	86	95
Muller-Lyer	ヒ (hi)	line	50	100
Muller-Lyer	ヒ (hi)	line	73	100
Hefler	サ (sa)	line	100	100
Hefler	サ (sa)	line	91	100
Hefler	キ (ki)	line	86	95
Hefler	キ (ki)	line	82	95
Zerbino	へ (he)	area	77	95
Zerbino	へ (he)	area	77	100
Ebbinghaus	カ (ka)	area	82	100
Ebbinghaus	カ (ka)	area	100	100
Jastrow	ト (to)	area	73	100
Jastrow	ト (to)	area	82	95
Delboeuf	フ (hu)	area	77	100
Delboeuf	フ (hu)	area	82	100
Ebbinghaus	ス (su)	area	59	100
Ebbinghaus	ス (su)	area	77	100
Ebbinghaus	ミ (mi)	area	86	100
Ebbinghaus	ミ (mi)	area	86	100
Jastrow	レ (re)	area	32	86
Jastrow	レ (re)	area	45	95

### fMRI data

#### Shape task vs. baseline

**Fig 3:**
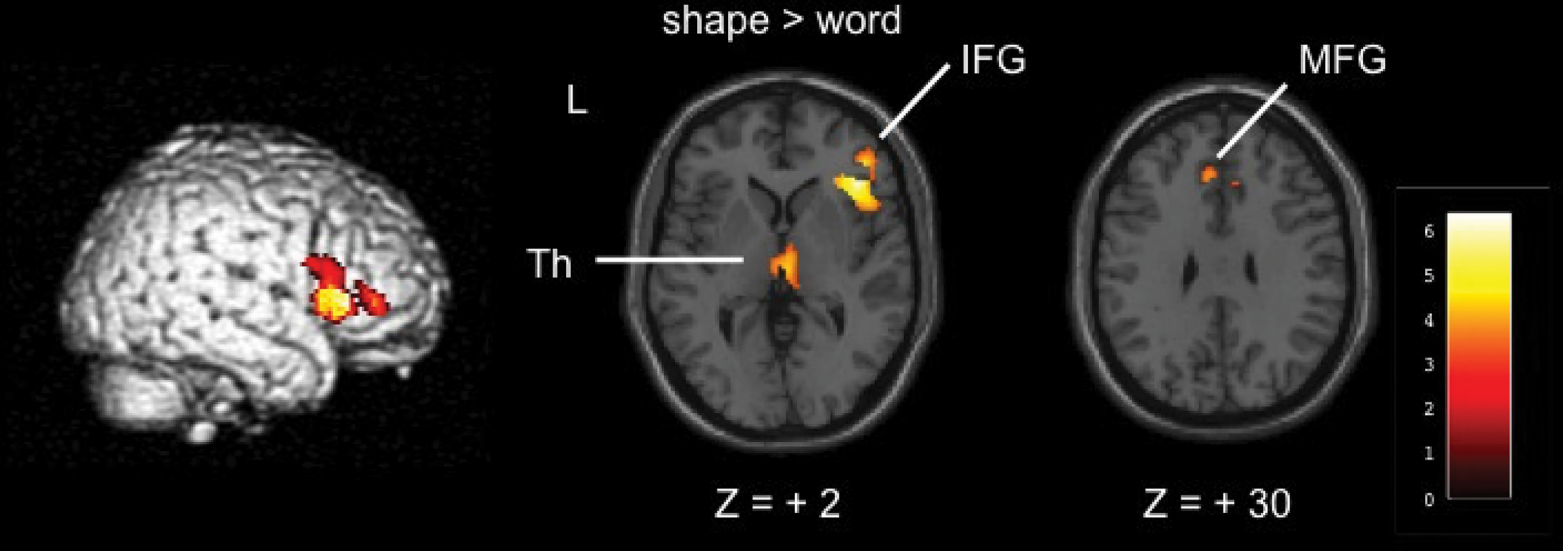
Shape task vs. baseline. a: shape > baseline (identified as optical illusions: *p* < 0.05, family-wise error [FWE] corrected), b: shape > baseline (not identified as optical illusions: *p* < 0.05, FWE-corrected). IFG = inferior frontal gyrus, MFG = middle frontal gyrus, Th = thalamus, MOG = middle occipital gyrus.

During the shape task, the trials in which stimuli were identified as optical illusions were associated with bilateral activation in the inferior and superior frontal gyrus, precentral gyrus, cingulate gyrus, thalamus (dorsomedial nucleus [dm]), ventral lateral nucleus [vl]), and the pulvinar [p]), cuneus, and occipital area relative to baseline (voxel-level corrected) (Fig 3A, Table 2). During the trials in which the stimuli were not identified as optical illusions, bilateral activation occurred in the inferior and superior frontal gyrus, precentral gyrus, cuneus, and occipital area relative to baseline (voxel-level corrected), but there was no bilateral activation in the thalamus, including the pulvinar (Fig 3B, Table 3).

**Table 2:** Trials in which stimuli were identified as optical illusions in the shape task compared to baseline.

Area	BA	Talairach coordinates (mm)			Z value	Cluster size in voxels
		x	y	z		
R. Inferior Frontal Gyrus	46	46	41	9	4.64	212
R. Inferior Frontal Gyrus	45	51	18	12	6.22	864
R. Inferior Frontal Gyrus	47	42	27	-3	5.82	
R. Superior Temporal Gyrus	22	48	11	-6	6.33	
R. Superior Frontal Gyrus	6	2	9	55	5.15	397
R. Cingulate Gyrus	32	2	12	36	5.08	
R. Thalamus (dm, vl, p)		12	-10	4	5.85	348
R. Middle Occipital Gyrus	18	12	-92	18	6.78	1285
R. Middle Occipital Gyrus	19	36	-87	15	6.35	
L. Inferior Frontal Gyrus	47	-38	27	-8	5.78	320
L. Precentral Gyrus	44	-51	12	5	5.19	
L. Superior Temporal Gyrus	22	-48	11	-6	6.06	
L. Thalamus (dm, vl, p)		-12	-17	3	4.98	169
L. Cuneus	18	-2	-96	23	6.55	962
L. Cuneus	19	-12	-90	30	5.98	
L. Middle Occipital Gyrus	19	-34	-83	4	6.25	380
L. Inferior Occipital Gyrus	18	-40	-84	-4	6.04	

Note: R. = right, L. = left, BA = Brodmann area, dm = dorsomedial nucleus, vl = ventral lateral nucleus, p = pulvinar.

**Table 3:** Trials in which stimuli were not identified as optical illusions compared to baseline.

Area	BA	Talairach coordinates (mm)			Z value	Cluster size in voxels
		x	y	z		
R. Superior Frontal Gyrus	8	4	24	50	5.75	1063
R. Superior Frontal Gyrus	6	4	7	59	5.64	
R. Inferior Frontal Gyrus	46	46	43	9	5.04	119
R. Inferior Frontal Gyrus	47	48	38	-14	5.50	963
R. Superior Temporal Gyrus	22	53	13	-4	6.00	
R. Middle Occipital Gyrus	18	12	-92	18	6.85	969
R. Middle Occipital Gyrus	19	36	-81	13	6.78	
L. Precentral Gyrus	44	-59	12	7	5.89	326
L. Superior Temporal Gyrus	22	-50	10	-2	6.27	
L. Superior Frontal Gyrus	6	-4	-1	63	5.36	1063
L. Frontal pole	10	-44	41	-4	5.11	114
L. Middle Occipital Gyrus	18	-24	-85	19	6.40	868
L. Cuneus	18	-14	-92	19	6.31	
L. Middle Occipital Gyrus	19	-34	-83	4	6.41	237

Note: R. = right, L. = left, BA = Brodmann area.

Furthermore, for trials in which stimuli were identified as optical illusions, we compared the data for the shape tasks with lines and areas to the baseline data. There was no difference among the types of stimuli.

#### Word task vs. baseline

For this analysis, we used the trials in which the stimuli were identified as words. Compared to the baseline, the word task induced bilateral activation in the inferior and superior frontal gyrus, cingulate gyrus, thalamus (dm, vl, and p), and occipital area (voxel-level corrected). In addition, activation was observed in the inferior parietal lobule including Brodmann area (BA) 40 (voxel-level corrected) in both tasks compared to the baseline.

Furthermore, for trials in which stimuli were identified as words, we compared the data for the word tasks with lines and areas to the baseline data. There was no difference among the types of stimuli.

#### Shape task vs. word task and word task vs. shape task

**Fig 4:**
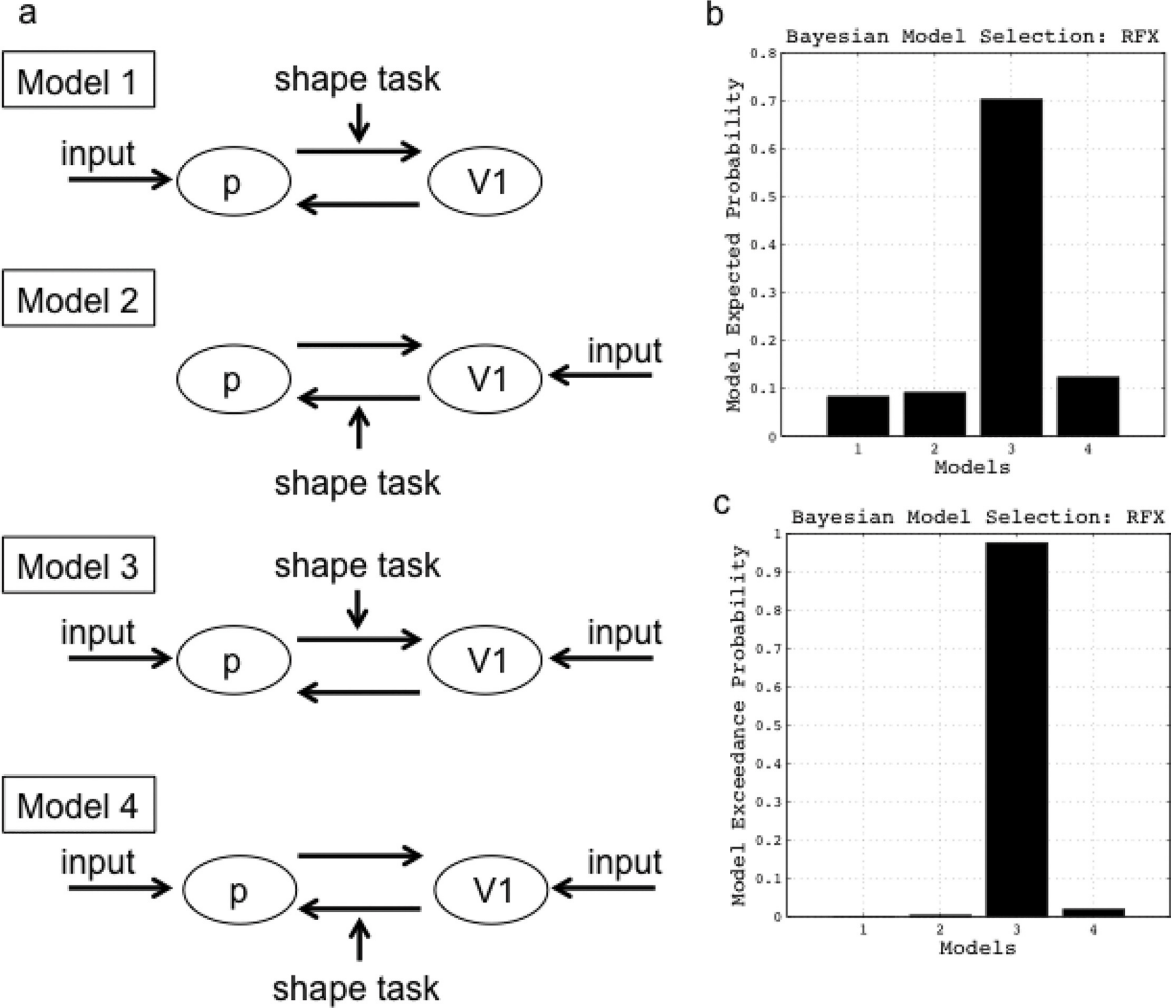
Shape task vs. word task (cluster threshold of *p* < 0.05 [corrected] with a voxel threshold of *p* < 0.001 [uncorrected]). Th = thalamus, IFG = inferior frontal gyrus, MFG = medial frontal gyrus.

We used the trials in which the stimuli were identified as optical illusions in the shape task or as words in the word task for these analyses. Therefore, the number of experimental trials was different in both tasks. The shape vs. word task comparison (Fig 4, Table 4) revealed activation in the right inferior frontal gyrus and left medial frontal gyrus, and in the right thalamus including the pulvinar (cluster-level corrected). In contrast, the word vs. shape task comparison did not reveal significant activation.

**Table 4:** Shape vs. word task using trials in which stimuli were identified as optical illusions in the shape task or as words in the word task.

Area	BA	Talairach coordinates (mm)			Z value	Cluster size in voxels
		x	y	z		
R. Inferior Frontal Gyrus	47	44	27	2	5.75	726
R. Inferior Frontal Gyrus	45	36	24	6	5.06	
R. Inferior Frontal Gyrus	46	46	35	2	4.37	
R. Thalamus (dm, vl, p)		2	-17	5	4.49	187
L. Medial Frontal Gyrus	9	-4	31	30	4.33	251

Note: R. = right, L. = left, BA = Brodmann area, dm = dorsomedial nucleus, vl = ventral lateral nucleus, p = pulvinar.

#### Effective connectivity

BMS was used for deciding which model best explained the measured responses. The values for the expected posterior probability were 0.08, 0.09, 0.7, and 0.12 in Models 1, 2, 3, and 4, respectively (Fig 2B). Model 3 out-performed the other three models, with an exceedance probability of 0.97 compared to 0.00, 0.01, and 0.02 in Models 1, 2, and 4, respectively (Fig 2C). The higher performance of Model 3 suggests that visual information was indeed input to V1 and the pulvinar, and that the shape task, as a modulatory input, had an effect on the forward connections from the pulvinar to V1.

## Discussion

We performed an fMRI study on healthy participants who viewed Müller-Lyer, Ponzo, Hefler, Zerbino, Ebbinghaus, Jastrow, and Delboeuf figures consisting of many Japanese letters. We used the same stimuli for the shape and word tasks. Our results demonstrated a large overlap in activation patterns when participants performed the shape and word tasks; the overlapping regions included the inferior and superior frontal gyrus, precentral gyrus, medial frontal gyrus, thalamus, and occipital area in both hemispheres. This pattern of commonly activated brain regions may constitute a shared neural network for processing both optical illusions and words; activation of the occipital area is involved in visual perception (e.g., [34]), that of the medial and inferior frontal gyrus is involved in resolving conflicting information (e.g., [35, 36]), and that of the precentral gyrus is involved in giving responses aloud (e.g., [37]).

Our results showed that line and area figures may have several types of neural processing in common for each task; the shape task with lines and areas vs. baseline comparison revealed activation in the inferior and superior frontal gyrus, precentral gyrus, insula, thalamus (dm, vl, and p), cuneus, and occipital area, whereas the word task with lines and areas vs. baseline comparison revealed activation in the inferior and superior frontal gyrus, cingulate gyrus, thalamus (dm, vl, and p), occipital area, and inferior parietal lobule. These results provide a new perspective on the neural mechanism of optical illusions.

The shape vs. word task comparison revealed activation in the dorsomedial nucleus, ventral lateral nucleus, and the pulvinar of the thalamus. The dorsomedial and ventral lateral nuclei of the thalamus are involved in activation of the prefrontal cortex and motor area, respectively [38–40]. The findings of the present study suggest that the pulvinar is involved in the cognitive processing of optical illusions. The optic pathway consists of both the geniculate and extrageniculate pathways, and the pulvinar is closely related to the extrageniculate system. The geniculate visual pathway follows a path from the retina to the lateral geniculate nucleus (LGN) and from there to the visual cortex. In contrast, the extrageniculate visual pathway consists of a pathway from the retina to the superior colliculus, the pulvinar, and finally the visual cortex. The pulvinar is considered the major source of visual processing during blindsight (e.g., [41, 42]), which is the ability to respond to visual stimuli without consciously seeing the stimulus and is exhibited by people with cortical blindness resulting from lesions in the primary visual cortex (Model 1 of Effective Connectivity Analysis).

Some variation in pulvinar activation across tasks was observed; it appeared strongest in the shape tasks, but was weaker in the word task. Only trials in which the stimuli were identified as optical illusions may demonstrate pulvinar activation. Thus, our results suggest that the extrageniculate system is associated with optical illusion processing. From the results of the effective connectivity analysis, the information from an optical illusion is projected to the visual cortex from both the LGN and pulvinar. We therefore may be able to detect optical illusions when visual information follows not only the geniculate but also the extrageniculate visual pathway.

Previous studies have suggested the involvement of feedback processing during the perception of an optical illusion; that is, an optical illusion may follow an inverse hierarchical path [23, 24, 27]. After extraction of global configural cues in the LOC, shape information may be sent back to the low-level areas in V1/V2. However, the feedback can also be explained by visual information input from the pulvinar. Thus, the time-lagged activation of V1/V2 after activation of the LOC demonstrated in previous studies may not reflect an inverse hierarchical path, but may rather indicate visual information input from the extrageniculate visual pathway. The suggestion that the extrageniculate visual pathway is involved in the perception of optical illusions is consistent with previous results indicating that mammals, birds, and insects can detect optical illusions [10, 43, 44].

Our results also demonstrated LOC activation during the shape task. Previous studies have suggested that the occipital cortex is the anatomical basis of optical illusions. In particular, the observation of LOC activation is consistent with the findings of previous studies [2, 3, 15–18]. The LOC is thought to be associated with the representation of objects, object fragments, and figure-ground segregation [45, 46]. Harris et al. [47] suggested that the LOC integrates local elements involved in perceiving an optical illusion. Vallar et al. [1] suggested that the anatomical basis of the Müller-Lyer illusion lies in regions dedicated to the processing of illusory contours. The present study not only confirms these previous findings but also extends them by showing LOC activation in a mixture of responses to various optical illusions other than Kanizsa-type and Müller-Lyer figures.

In addition, we found activation of the frontal lobes during the shape task. The activation of the frontal lobes was stronger in the right hemisphere than in the left hemisphere. This is in agreement with electroencephalography (EEG) and magnetoencephalography (MEG) data reported by Qiu et al. [48] and Weidner et al. [3], respectively, which suggested that this component reflects high-level cognitive control related to judging information from the illusion. This suggestion agrees with data reported by Vossel et al. [49], who found that the right posterior inferior frontal gyrus is activated by stimulus changes in an oddball task. Therefore, we suggest that activation of the right inferior frontal gyrus was involved in judging whether the two stimuli were identical during our experiment.

The present study has the following limitations. First, the time course of perceiving an optical illusion was not clarified. In contrast to hemodynamic imaging methods, EEG and MEG provide a finer temporal analysis of the time course of the processes involved in the perception of an optical illusion, although their spatial resolution is much less reliable than that of positron emission tomography or fMRI. Second, we used the same stimuli consisting of many kana letters for the shape and word tasks. Therefore, our optical illusion stimuli were different from the original figure images. The relatively low identification rate for the optical illusion stimuli might have been caused by this difference. However, in our analysis, we used only trials in which stimuli were identified as optical illusions; therefore, the low identification rate may not have influenced our results. Third, differences in task difficulty between the shape and word tasks might have affected the results. For example, the results might represent a stronger decision conflict in the shape task relative to the word task. Therefore, there may have been less reliable information available for the shape task, which may have thus resulted in “noisier” data. However, in our analysis, during the shape task, the trials in which stimuli were identified as optical illusions were associated with the pulvinar. During the trials in which the stimuli were not identified as optical illusions, there was no bilateral activation in the thalamus, including the pulvinar. These results may indicate that the extrageniculate system is involved in the cognitive processing of optical illusions without any relationship to the difficulty of the shape and word tasks. In the future, some adjustments of the difficulty between the shape and word tasks will be required to eliminate the decision conflict factor.

## References

[1] Vallar G, Daini R, Antonucci G. Processing of illusion of length in spatial hemineglect: a study of line bisection. Neuropsychologia. 2000;38(7):1087–97. 10775718 10.1016/s0028-3932(99)00139-6

[2] Weidner R, Fink GR. The neural mechanisms underlying the Muller-Lyer illusion and its interaction with visuospatial judgments. Cereb Cortex. 2007;17(4):878–84. 16707733 10.1093/cercor/bhk042

[3] Weidner R, Boers F, Mathiak K, Dammers J, Fink GR. The temporal dynamics of the Muller-Lyer illusion. Cereb Cortex. 2010;20(7):1586–95. 10.1093/cercor/bhp217 19875676

[4] Bruno N, Bernardis P, Gentilucci M. Visually guided pointing, the Muller-Lyer illusion, and the functional interpretation of the dorsal-ventral split: conclusions from 33 independent studies. Neurosci Biobehav Rev. 2008;32(3):423–37. 17976722 10.1016/j.neubiorev.2007.08.006

[5] Shapley R, Maertens M. Angle alignment evokes perceived depth and illusory surfaces. Perception. 2008;37(10):1471–87. 19065852 10.1068/p5987 PMC3063121

[6] Underleider LG, Mishkin M. Two cortical visual systems In: Ingle DJ, Goodale MA, and Mansfield RJW, editors. Analysis of visual behavior. Cambridge: MIT Press; 1982 pp. 549–586.

[7] Goodale MA, Milner AD. Separate visual pathways for perception and action. Trends Neurosci. 1992;15(1):20–5. 1374953 10.1016/0166-2236(92)90344-8

[8] Lyon DC, Nassi JJ, Callaway EM. A disynaptic relay from superior colliculus to dorsal stream visual cortex in macaque monkey. Neuron. 2010;65(2):270–9. 10.1016/j.neuron.2010.01.003 20152132 PMC2832737

[9] von der Heydt R, Peterhans E. Mechanisms of contour perception in monkey visual cortex. I. Lines of pattern discontinuity. J Neurosci. 1989;9(5):1731–48. 2723747 10.1523/JNEUROSCI.09-05-01731.1989 PMC6569817

[10] Ramsden BM, Hung CP, Roe AW. Real and illusory contour processing in area V1 of the primate: a cortical balancing act. Cereb Cortex. 2001;11(7):648–65. 11415967 10.1093/cercor/11.7.648

[11] Sasaki Y, Watanabe T. The primary visual cortex fills in color. Proc Natl Acad Sci U S A. 2004;101(52):18251–6. 15596726 10.1073/pnas.0406293102 PMC539772

[12] Murray SO, Boyaci H, Kersten D. The representation of perceived angular size in human primary visual cortex. Nat Neurosci. 2006;9(3):429–34. 16462737 10.1038/nn1641

[13] Fang F, Boyaci H, Kersten D, Murray SO. Attention-dependent representation of a size illusion in human V1. Curr Biol. 2008;18(21):1707–12. 10.1016/j.cub.2008.09.025 18993076 PMC2638992

[14] Sperandio I, Chouinard PA, Goodale MA. Retinotopic activity in V1 reflects the perceived and not the retinal size of an afterimage. Nat Neurosci. 2012;15(4):540–2. 10.1038/nn.3069 22406550

[15] Hirsch J, DeLaPaz RL, Relkin NR, Victor J, Kim K, Li T, et al Illusory contours activate specific regions in human visual cortex: evidence from functional magnetic resonance imaging. Proc Natl Acad Sci USA. 1995;92(14):6469–73. 7604015 10.1073/pnas.92.14.6469 PMC41539

[16] Seghier M, Dojat M, Delon-Martin C, Rubin C, Warnking J, Segebarth C, et al Moving illusory contours activate primary visual cortex: an fMRI study. Cereb Cortex. 2000;10(7):663–70. 10906313 10.1093/cercor/10.7.663 PMC2737131

[17] Ritzl A, Marshall JC, Weiss PH, Zafiris O, Shah NJ, Zilles K, et al Functional anatomy and differential time courses of neural processing for explicit, inferred, and illusory contours. An event-related fMRI study. Neuroimage. 2003;19(4):1567–77. 12948712 10.1016/s1053-8119(03)00180-0

[18] Brighina F, Ricci R, Piazza A, Scalia S, Giglia G, Fierro B. Illusory contours and specific regions of human extrastriate cortex: evidence from rTMS. Eur J Neurosci. 2003;17(11):2469–74. 12814379 10.1046/j.1460-9568.2003.02679.x

[19] Daini R, Angelelli P, Antonucci G, Cappa SF, Vallar G. Exploring the syndrome of spatial unilateral neglect through an illusion of length. Exp Brain Res. 2002;144(2):224–37. 12012160 10.1007/s00221-002-1034-8

[20] Spillmann L, Dresp B. Phenomena of illusory form: can we bridge the gap between levels of explanation? Perception. 1995;24(11):1333–64. 8643336 10.1068/p241333

[21] Watt SJ, Bradshaw MF, Rushton SK. Field of view affects reaching, not grasping. Exp Brain Res. 2000;135(3):411–6. 11146819 10.1007/s002210000545

[22] Zeki S. The neurology of ambiguity. Conscious Cogn. 2004;13(1):173–96. 14990252 10.1016/j.concog.2003.10.003

[23] Rao RP, Ballard DH. Predictive coding in the visual cortex: a functional interpretation of some extra-classical receptive-field effects. Nat Neurosci. 1999;2(1):79–87. 10195184 10.1038/4580

[24] Hochstein S, Ahissar M. View from the top: hierarchies and reverse hierarchies in the visual system. Neuron. 2002;36(5):791–804. 12467584 10.1016/s0896-6273(02)01091-7

[25] Murray MM, Wylie GR, Higgins BA, Javitt DC, Schroeder CE, Foxe JJ. The spatiotemporal dynamics of illusory contour processing: combined high-density electrical mapping, source analysis, and functional magnetic resonance imaging. J Neurosci. 2002;22(12):5055–73. 12077201 10.1523/JNEUROSCI.22-12-05055.2002 PMC6757726

[26] Halgren E, Mendola J, Chong CD, Dale AM. Cortical activation to illusory shapes as measured with magnetoencephalography. Neuroimage. 2003;18(4):1001–9. 12725774 10.1016/s1053-8119(03)00045-4

[27] Lamme VA. Why visual attention and awareness are different. Trends Cogn Sci (Regul Ed). 2003;7(1):12–8. 12517353 10.1016/s1364-6613(02)00013-x

[28] Wokke ME, Vandenbroucke AR, Scholte HS, Lamme VA. Confuse your illusion: feedback to early visual cortex contributes to perceptual completion. Psychol Sci. 2013;24(1):63–71. 10.1177/0956797612449175 23228938

[29] Shiina K. Psychology of illusion [in Japanese]. Tokyo: Kodansha; 1995.

[30] Baba Y, Tanaka Y. Assent illusion Figure Scripture to try!—Whole experience to new discoveries from classics! [in Japanese]. Tokyo: Kodansha; 2004.

[31] Talairach J, Tournoux P. Co-Planar Stereotaxic Atlas of the Human Brain. New York: Thieme Medical Publishers; 1988.

[32] Lancaster JL, Woldorff MG, Parsons LM, Liotti M, Freitas CS, Rainey L, et al Automated Talairach atlas labels for functional brain mapping. Hum Brain Mapp. 2000;10(3):120–31. 10912591 10.1002/1097-0193(200007)10:3<120::AID-HBM30>3.0.CO;2-8 PMC6871915

[33] Friston KJ, Harrison L, Penny W. Dynamic causal modelling. Neuroimage. 2003;19(4):1273–302. 12948688 10.1016/s1053-8119(03)00202-7

[34] Hubel DH, Wiesel TN. Ferrier lecture. Functional architecture of macaque monkey visual cortex. Proc R Soc Lond, B, Biol Sci. 1977;198(1130):1–59. 20635 10.1098/rspb.1977.0085

[35] Kemmotsu N, Villalobos ME, Gaffrey MS, Courchesne E, Müller R-AA. Activity and functional connectivity of inferior frontal cortex associated with response conflict. Brain Res Cogn Brain Res. 2005;24(2):335–42. 15993771 10.1016/j.cogbrainres.2005.02.015

[36] West R, Bowry R, McConville C. Sensitivity of medial frontal cortex to response and nonresponse conflict. Psychophysiology. 2004;41(5):739–48. 15318880 10.1111/j.1469-8986.2004.00205.x

[37] Tremblay P, Gracco VL. Contribution of the frontal lobe to externally and internally specified verbal responses: fMRI evidence. Neuroimage. 2006;33(3):947–57. 16990015 10.1016/j.neuroimage.2006.07.041

[38] Tobias TJ. Afferents to prefrontal cortex from the thalamic mediodorsal nucleus in the rhesus monkey. Brain Res. 1975;83(2):191–212. 1109293 10.1016/0006-8993(75)90930-0

[39] Schell GR, Strick PL. The origin of thalamic inputs to the arcuate premotor and supplementary motor areas. J Neurosci. 1984;4(2):539–60. 6199485 10.1523/JNEUROSCI.04-02-00539.1984 PMC6564912

[40] Strick PL. Light microscopic analysis of the cortical projection of the thalamic ventrolateral nucleus in the cat. Brain Res. 1973;55(1):1–24. 4713188 10.1016/0006-8993(73)90485-x

[41] Brindley GS, Gautier-Smith PC, Lewin W. Cortical blindness and the functions of the non-geniculate fibres of the optic tracts. J Neurol Neurosurg Psychiatr. 1969;32(4):259–64. 5807867 10.1136/jnnp.32.4.259 PMC496510

[42] Cowey A. The blindsight saga. Exp Brain Res. 2010;200(1):3–24. 10.1007/s00221-009-1914-2 19568736

[43] Nieder A. Seeing more than meets the eye: processing of illusory contours in animals. J Comp Physiol A Neuroethol Sens Neural Behav Physiol. 2002;188(4):249–60. 12012096 10.1007/s00359-002-0306-x

[44] Pan Y, Chen M, Yin J, An X, Zhang X, Lu Y, et al Equivalent representation of real and illusory contours in macaque V4. J Neurosci. 2012;32(20):6760–70. 10.1523/JNEUROSCI.6140-11.2012 22593046 PMC6622189

[45] Grill-Spector K, Kourtzi Z, Kanwisher N. The lateral occipital complex and its role in object recognition. Vision Res. 2001;41(10–11):1409–22. 11322983 10.1016/s0042-6989(01)00073-6

[46] Konen CS, Kastner S. Two hierarchically organized neural systems for object information in human visual cortex. Nat Neurosci. 2008;11(2):224–31. 10.1038/nn2036 18193041

[47] Harris JJ, Schwarzkopf DS, Song C, Bahrami B, Rees G. Contextual illusions reveal the limit of unconscious visual processing. Psychol Sci. 2011;22(3):399–405. 10.1177/0956797611399293 21317371 PMC3278746

[48] Qiu FT, von der Heydt R. Figure and ground in the visual cortex: v2 combines stereoscopic cues with gestalt rules. Neuron. 2005;47(1):155–66. 15996555 10.1016/j.neuron.2005.05.028 PMC1564069

[49] Vossel S, Weidner R, Thiel CM, Fink GR. What is “odd” in Posner’s location-cueing paradigm? Neural responses to unexpected location and feature changes compared. J Cogn Neurosci. 2009;21(1):30–41. 10.1162/jocn.2009.21003 18476756

